# A retrospective analysis of the health and social situation of homeless people in Berlin: study protocol

**DOI:** 10.1186/s13690-021-00546-y

**Published:** 2021-03-06

**Authors:** Sonia Lech, Liane Schenk, Jenny De la Torre Castro, Daniel Schindel

**Affiliations:** 1 Charité – Universitätsmedizin Berlin, corporate member of Freie Universität Berlin, Humboldt-Universität zu Berlin, and Berlin Institute of Health, Institute of Medical Sociology and Rehabilitation Science, Berlin, Germany; 2 Jenny De la Torre Foundation, Berlin, Germany

**Keywords:** Homeless, Social deprivation, Health, Medical records, Longitudinal data

## Abstract

**Background:**

Homelessness is often described as both a driver and a consequence of poor health, social exclusion and economic marginalisation. The present protocol provides a detailed description of the study *Examining the health situation of homeless people in Berlin: a retrospective analysis of data from the health centre for the homeless of the Jenny De la Torre Foundation from 2006 to 2020* (GIG study). The primary objective of the GIG study is to describe and analyse the social and health situation of homeless people in Berlin.

**Methods:**

A retrospective secondary data analysis of an anonymous full census of medical records for the years 2006 until 2020 from a health centre for homeless people will be carried out. The main outcome is the description and analysis of the social and health situation of homeless people in Berlin. Total and cross-sectional sample characteristics will be presented in a descriptive analysis using Chi-Square Test, Mann-Whitney-U-Test or independent t-Test as appropriate to test (sub) group differences. Further, outcomes will be analysed using finite mixture modelling in order to distinguish different types of social and health conditions. Latent variable regressions will be applied in order to identify sociodemographic and disease-related factors associated with decreasing health conditions.

**Discussion:**

Given the high number of homeless individuals in Germany, it is of great importance to examine their social and health situation in order to gain a better understanding of challenges and needs of homeless people and work on new approaches and solutions to effectively address these.

**Trail registration:**

The study was prospectively registered with the German Clinical Trials Register (trial registration number: DRKS00021172). Registered 26 June 2020.

## Background

Homelessness is an extreme reflection of poverty and social exclusion [1] and represents a complex social and public health challenge [2]. Previous research has acknowledged the difficulty in the assessment of the scale of homelessness across Europe, due to inconsistencies in the definitions of homelessness as well as the variety of methodologies applied in data collection [3, 4]. In 2018 about 678,000 homeless people were living in Germany out of which approximately 41,000 spent at least some of their nights without shelter on the streets [5]. In Berlin, 60,000 people are currently estimated to be homeless [6] of which 2000–6000 sleeping rough [7, 8].

Homelessness is often described as both a driver and a consequence of poor health, social exclusion and economic marginalisation [9–11]. Life on the street or in collective accommodation, shelters or hostels represents a major health burden and is often associated with a variety of health problems [12]. For example, research depicts the consistently high rates of acute infectious and parasitic diseases, diseases of the circulatory, respiratory and musculoskeletal system [13] as well as high rates of blood borne infections such as hepatitis C, HIV, tuberculosis, as well as myocardial infarction and pneumonia [14–16]. In addition to acute conditions, a high percentage of the homeless suffer from multimorbidity and chronic diseases [14, 17] such as chronic pain [18] or chronic obstructive pulmonary disease [19]. A study by Queen, Lowrie, Richardson & Williamson [20] found in a homeless cohort with an average age of 42.8 years comparable levels of multimorbidity with those aged > 85 years in the general population. High prevalence of somatic diseases and harsh living conditions might also explain why homeless people generally suffer from higher mortality rates and earlier deaths [11, 21–23]. Homelessness is also associated with a higher risk of psychiatric disorders such as psychosis, major depression, personality disorders, alcohol dependency and drug dependency [24–26]. The high prevalence of somatic and psychiatric diseases can partially be explained by the various stressors associated with the loss of accommodation such as inadequate health maintenance, risk-prone health behaviours [27] and childhood traumata [28]. There have been a number of studies reporting high rates of smoking [29], as well as high rates of alcohol and/or substance misuse [30, 31]. Additionally, homeless people suffer from food insecurity resulting in a generally poorer diet characterised by higher intakes of salt and lower intakes of fruit, vitamin C and fibre [32]. Another major risk factor is the high exposure to violence and higher chance of injury experienced by homeless individuals [33–35]. Furthermore, medical care is usually provided outside the structures of the regular health care system and not always accessible [13, 36]. Rough sleepers are less likely to be registered with a general practitioner [37] and often do not have access to preventive care such as routine check-ups [38, 39]. The consequence of this lack of access to primary care results in a higher number of emergency department visits, hospital (re) admissions and longer inpatient stays [36, 40, 41]. This aspect is also reflected by the high use of emergency departments for dental problems by homeless people [42, 43].

To sum up, past research acknowledges a variety of health needs among homeless individuals. Despite growing recent evidence on the health status of homeless people, data and results do not allow for comparison across countries. Research on homelessness across the EU suffers from a variety of difficulties due to differences in definitions of homelessness and inconsistencies in applied research methodologies [3, 44–47]. Additionally, for Germany, there is a clear lack of empirical investigations on the social and health situation of homeless people. Further, homeless people are difficult to sample which limits most research and results in small and highly selected samples. A recent review found that previous research is highly selective were participants are often older, male and from a subgroup setting as for example from supervised drug consumption facilities [48]. Thus, there is a clear lack of investigation among women and younger individuals. In response to sampling difficulties, other countries such as Denmark, implemented a Homeless Register, where every contact in homeless shelters is documented [21]. There is a need of unbiased research of the homeless population in Germany. In addition, there is little evidence about the course of the social and health situation of homeless people. It is of great importance to gain better understanding of the social and health situation and needs of homeless people. The project  *Examining the health situation of homeless people in Berlin: a retrospective analysis of data from the health centre for the homeless of the Jenny De la Torre Foundation from 2006 to 2020 *(GIG study) aims to explore cross-sectional and longitudinal data from medical records of homeless individuals in Berlin and provide further insights on that subject’s matter. The objective of the present study is to present a study protocol and to describe potential limitations.

### Aim of the present study

The main aim of the present study protocol is the description of the objectives, design, methodology and potential limitations of the GIG study. The objectives of the retrospective analysis can be summarised as the following: 1) comprehensive socio-demographic characterisation of patients and 2) detailed description and analysis of health status and medical history (including utilisation of social and medical services). Both are analysed cross-sectional and longitudinal. Evaluation of longitudinal data will include analysis of individual courses (intra-individually for patients with multiple measure points), as well as analysis of trends where cross sections are considered over time (inter-individual analysis). In line with the study design (retrospective analysis of medical records), research questions will be examined in an explorative manner, based on type and quality of data available from medical records (document analysis). Among others, we aim to address the following research questions:

#### Objective 1

*O1a: Characterise the sample by age, education, gender, migration background, insurance status, detention experience, type of accommodation.*

*O1b: How often is there a change in the homeless status over time? How does the socio-demographic structure change over time?*

#### Objective 2

*O2a: Which are the most frequent health problems homeless people face?*

*O2b: How is the health and social services utilisation among homeless people?*

*O2c: How often/long are patients receiving care in the health centre?*

*O2d: Is there a change in health or social challenges during the last years?*

*O2e: Is there a change in frequency of specific diseases over time?*

*O2f: Are there associations between extreme social conditions and the health status of individuals?*

## Methods

### Study design

**Fig. 1:**
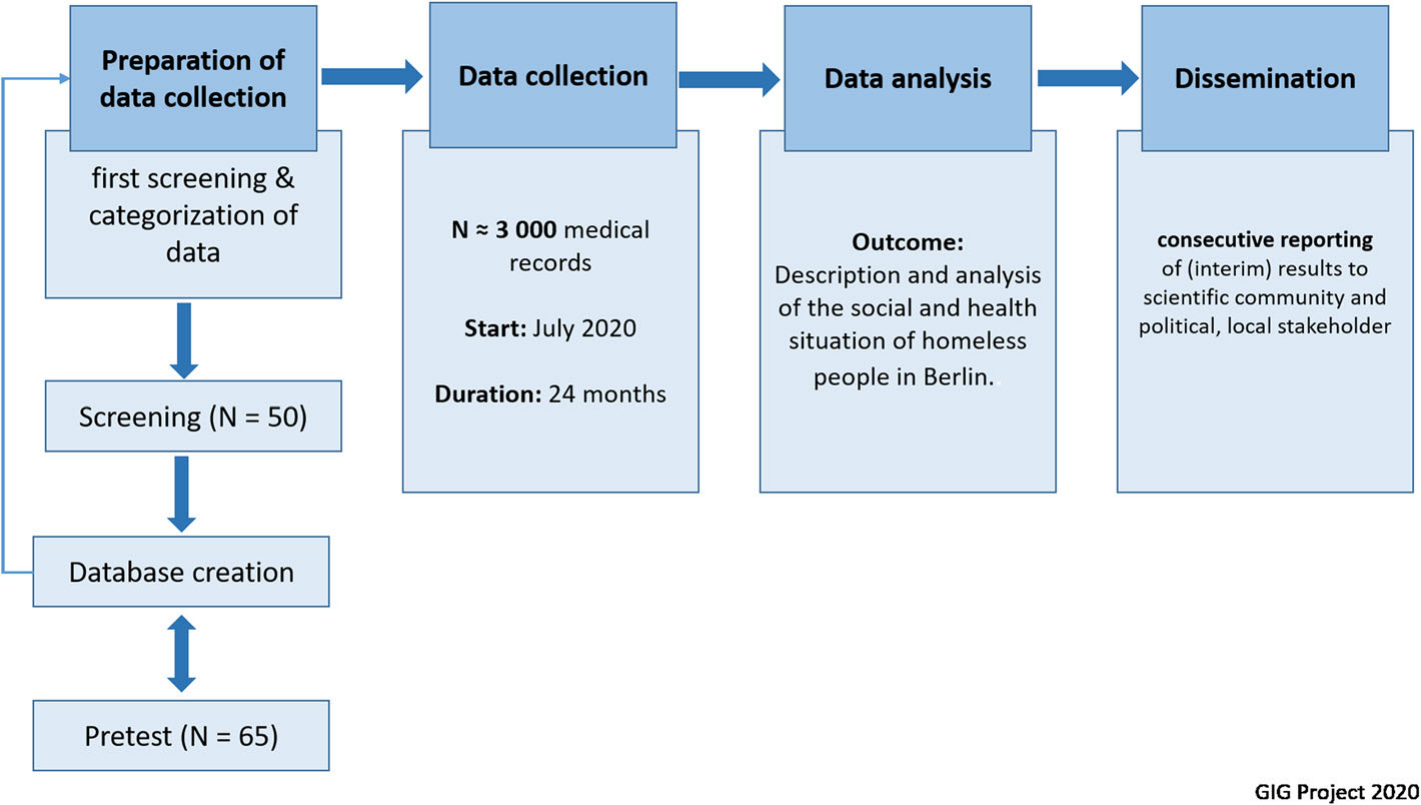
Overview of the study design

To explore the social and health situation of homeless people in Berlin a retrospective analysis of medical records from the health centre for homeless people of the Jenny De la Torre Foundation will be carried out. An overview of the study design can be obtained from Fig. 1.

### Study setting

Data will be obtained from medical records collected and provided by the health centre for homeless people. The health centre provides donation-based and low-threshold medical care for homeless people. The centre includes a medical practice with medical specialists from various areas, a dental practice and an ophthalmic practice. Further, the centre offers psychological and social counselling as well as legal advice within various fields. In addition, two meals per day, clothing and a hairdresser are at the disposal of patients. The centre also offers the service of taking passport photographs. Every individual visiting the health centre has to first check in with the doctor’s office. At the first visit a medical record is generated for each person. Every time individuals visit the health centre, the reason of the visit and utilisation of any service is documented in the medical record. If required, patients may be treated anonymously.

### Database and data extraction

Based on the general structure and type of data available from medical records, the main purpose of this step was to build a database structure that allows extracting and entering as much relevant data as possible. Therefore, as part of a preliminary screening, *N* = 50 randomly selected medical records were screened for content and data availability. Groups and variables are designed under consideration of previous research [49, 50]. As a result, we identified eight different data sources that can be extracted from medical records (see Table 1): 1) *floating medical record* (a file that contains all information available), 2) *social and medical anamnesis* (social and medical history of the patient; a standardised form that is filled out by the medical personnel at the first visit), 3) *patient information sheet* (a standardised form that contains information on any change of the homelessness, health insurance status, as well as the receipt of financial support), 4) *doctors letters* (including reports and hospital discharge letters), 5) *psychological report* (patients who make use of the psychological counselling in the centre are documented and a report of the counselling is written by the psychologist), 6) *psychiatric record* (a medical record about the psychological well-being of a patient), 7) *documents provided by the health centre* (doctors certificates or sick certificates), and 8) *others* (any other data available from the medical record). Based on the structure of medical records, a database was created using the software Research Electronic Data Capture (REDCap). REDCap is a browser-based, metadata-driven EDC software for designing research databases. It is secured under data protection law by the Charité – Universitätsmedizin Berlin. The database is accessible online which simplifies the entry of data on site while ensuring data safety and protection. No personal identifying data is collected. During data collection, the survey is pseudonymised. For analysis and presentation of results, anonymised data is used. Data extraction started in July 2020 and will end in July 2022.

**Table 1:** Overview of different data sources

***data source***		***topic***	***variables***	***categories***	***related objectives/ research questions***
**1**	**Floating medical record**	General information	status of homelessness/ type of accommodation	own flat, hostel, sleeps at friends/ acquaintances, shelter, carrier housing, sleeping rough, other	O1a, O1b, O2d
			status of health insurance	not insured, insured (type of company)	O1a, O1b, O2d
			birthday	year of birth	O1a, O2d
			number of visits	in total	O1a, O1b, O2d
			number of medical consultations	–	O1a, O1b, O2d
			duration of care (date of first and last visit)	in years	O1a, O1b, O2d
			indications of debts	no, yes	O1a, O1b, O2d
		Utilization of health centre services	medical reason	need of primary care, eye specialist, dentist	O2b, O2c
			counselling	(at risk of) homelessness in need of psychologist, social worker, legal advice	O2b, O2c
			Other	food, new clothes, hairdresser, shower, other	O2b, O2c
		Medical history	diagnosis and provided treatment, medication, further recommendations	date of issue, 3-digit ICD-10-GM code*, prescribed medication by ATC code**	O2a, O2b, O2c, O2d, O2e, O2f
**2**	**Social and medical anamnesis**	Socio demographics	age	in years	O1a, O2d, O2f
			sex	female, male, diverse	O1a, O2d, O2f
			school education	number of school years	O1a, O2d, O2f
			occupational education	unskilled, three-year apprenticeship, technical college degree, university degree	O1a, O2d, O2f
			citizenship	list of 202 countries	O1a, O2d, O2f
			family status	married (living together), married (living apart), single, divorced, widowed	O1a, O1b, O2d, O2f
			family contact	no, yes (parents, siblings, children, other)	O1a, O1b, O2d, O2f
			(un)employment	years of unemployment	O1a, O1b, O2d, O2f
			reason for homelessness	job loss, divorce, debt, mental illness, change of location, detention, rental rate, escape from parental home, lease expired, other	O1a, O1b, O2d, O2f
			source of income	social assistance, basic security, unemployment benefit, pension, newspaper sale, collect bottles, begging, other	O1a, O1b, O2d, O2f
			detention experiences	no, yes	O1a, O1b, O2d, O2f
		Medical history	somatic diseases	3-digit ICD-10-GM code*, date of consultation	O2a, O2d, O2e, O2f
			psychiatric diseases	3-digit ICD-10-GM code* (F00-F99), date of consultation	O2a, O2d, O2e, O2f
			dental health	dental problems, consultation of dentist, date of consultation	O2a, O2d, O2e, O2f
			infectious diseases	hepatitis A, B, C, HIV, sexually transmitted diseases	O2a, O2d, O2e, O2f
			medication	prescribed medication by ATC code**, date of prescription	O2a, O2d, O2e, O2f
**3**	**Patient information sheet**	Change of general information	status of homelessness/ type of accommodation	own flat, hostel, sleeps at friends/ acquaintances, shelter, carrier housing, sleeping rough, other	O1b, O2d, O2f
			status of health insurance	not insured, insured (type of company)	O1b, O2d, O2f
			status of financial support	social assistance, basic security, unemployment benefit, pension, other	O1b, O2d, O2f
**4**	**Doctors letters**	Hospital care	hospital discharge letters	institution, date of admission and discharge, date of issue, 3-digit ICD-10-GM code*, prescribed medication by ATC code**	O1a, O2a, O2b
		Ambulatory care	physician’s letter	date of issue, 3-digit ICD-10-GM code*, prescribed medication by ATC code**	O1a, O2a, O2b
**5**	**Psychological report**	Psychological counselling	reason for counselling	date of issue, agreements (appointment: with social worker, for legal advice, with psychologist, job centre, other)	O1a, O2a, O2b
			diagnosis	3-digit ICD-10-GM code* (F00-F99)	O1a, O2a, O2b
**6**	**Psychiatric record**	Psychiatric record	diagnosis	date of issue, 3-digit ICD-10-GM code*	O1a, O2a, O2b
			medication	prescribed medication by ATC code**	O1a, O2a, O2b
**7**	**Documents provided by the health centre**	Certificates	doctors’ certificates	issued for which institution, date of issue, 3-digit ICD-10-GM code*, prescribed medication by ATC code**	O1a, O2a, O2b
		Reports	any report	issued for which institution, date of issue, 3-digit ICD-10-GM code*, prescribed medication by ATC code**	O1a, O2a, O2b
**8**	**Others**	Other information provided in the medical record	x-ray images, electrocardiogram recordings, pictures, letters, notes, other documents	existing yes, no	O1a, O2a, O2b, O2c, O2f

*ICD-10-GM International Classification of Diseases German Modification, **ATC code Anatomical Therapeutic Chemical Classification System

### Inclusion and exclusion criteria

Every medical record created in the health centre for homeless people from the year 2006 until 2020 will be entered in the database and included in the analysis (N ≈ 3000). There are no other inclusion or exclusion criteria.

### Measures

Data will be extracted from medical records and was collected within routine medical care by medical doctors and health care personnel. Data was primarily collected for the purpose of medical care documentation in the health centre. However, a variety of health and social information can be obtained from the medical records. Generally, data can be divided in eight data sources. An overview of all data sources can be obtained from Table 1.

### Pretest and quality assurance

After creating a first database, a pretest was conducted to ensure a complete and fully operational data entry. The pretest included *N* = 55 medical records. Data was entered by two experienced research associates. Data entry was documented and conspicuous differences and needs for adjustment collected. Based on the pretest the database was revised and edited. The new database was again tested with *N* = 10 medical records. Further, minor changes where implemented, and the data base was finalised. Data will be entered by two trained medical students. For quality insurance, prior to the beginning of data entry, a training on the data base and data collection was conducted with the medical students. Further, a code book for data entry was developed. Regular supervisions and random controls of data entry will be taking place during the entire period of data entry.

### Statistical analysis

Total and cross-sectional sample characteristics will be presented in a descriptive analysis using Chi-Square Test, Mann-Whitney-U-Test or independent t-Test as appropriate to test (sub) group differences. The absolute standardised mean difference (ASMD) will be calculated to check the balancing of the characteristics. We consider ASMD< 0.1 as adequate balance between groups. The level of significance will be considered at 0.05. Further, statistical analyses might use finite mixture modelling – particularly latent class analysis – in order to distinguish different types of social and health conditions. We will conduct latent variable regression in order to identify sociodemographic and disease-related factors associated with decreasing health conditions.

By performing latent class analysis collinearity between indicator variables is not a problem, since these procedures are aimed precisely at such configurations and patterns. Latent class analysis explicitly assumes that manifest variables occur in common expressions. In contrast, the usual limitations of conventional regression models apply for exogenous covariates. To increase estimation accuracy, we aim at testing for collinearity between covariates using the following two different approaches. First, a correlation matrix of predictor variables will be calculated. Bivariate correlations larger 0.8 indicate collinearity. Second, we will compute coefficients of determination of each independent variable regressed on the remaining predictor variables to reveal collinear relationships involving more than two variables. Single coefficients of determination being larger than the overall model’s coefficient of determination indicate collinearity [51].

All statistical analyses will be performed using IBM SPSS statistics software (IBM SPSS Statistics for Windows, Version 25.0).

### Program governances and ethical approval

The research project will be conducted according to the principles of Good Clinical Practice and the Declaration of Helsinki and was prospectively registered with the German Clinical Trials Register (trial registration number: DRKS00021172). The study received Human Research Ethics Committee approval from Charité – Universitätsmedizin Berlin (EA1/058/20). Further, the GIG study established an independently chaired steering committee prior to the start of data entry. It is comprised of representatives, program funders, board members, service providers engaged in the program, state government and the lead Chief Investigator of the research team. The committee members receive on a regular base short briefings from the research team on the current state of the project and in return, give advice on the research project. Additionally, the committee meets once a year in order to present and discuss the current state of the project. The first meeting already took place via a conference call in June 2020. The next chaired steering meeting will take place in June 2021.

## Discussion

Given the number of homeless individuals in Germany, it is of great importance to explore the social and health situation of homeless people in order to better understand challenges and needs as well as work on new health care solution approaches. The present study addresses an important gap in literature as data on this matter is lacking, especially in Germany. The present study has substantial strengths including a secondary database which contains information on the health and social situation of homeless people collected in a health centre for homeless people under naturalistic conditions. Medical record data represent a unique possibility for a better understanding of the health situation and needs of homeless patients. The analysis will be exclusively based on medical record data. However, some medical records are incomplete whereas others are very exhaustive and contain lots of information including material that is not directly related to our research question (for example private pictures or letters of the patients). Another strength includes the study design, as longitudinal data is available for a long period of time (14 years) and for a large, representative population (about 3000 medical records). As no inclusion criteria are applied, a broad range of social and health situations will be analysed, including marginalised groups in research such as women and young patients [48]. Further, a big strength of the present study is the consideration of the course of time for both cross sectional analysis (analysis of trends) and longitudinal analysis (inter and intra individually) of data. This also enables a morbidity analysis. At the very minimum, the compilation and structuring of medical and social information by building categories in order to create an exploratory data base may contribute to the development of standardised instruments in the field of homelessness research [52].

To sum up, homelessness has serious implications for the health of individuals and populations. Based on the findings of the present study we expect to gain a better understanding on the health situation of homeless people in Germany and draw implications for its improvement by adapting services to changes in population demographics and morbidities. In order to improve health care services for homeless people, the further development of multifarious and diverse approaches seem necessary [53]. Thus, we expect the results to reveal a wide range of implications. For example, in a study conducted by Kaduszkiewicz et al. [13] a variety of concrete suggestions such as improving state funding and the range of health services and providing intermediate care centres were discussed. In order to achieve a long-term improvement, we believe it is inevitable to include and examine social policy and structural factors that contribute to or result in homelessness [54].

### Limitations

A number of limitations have to be acknowledged. First, the present study is based on a mono-centred survey in the centre of Berlin and therefore under risk of selection bias. The infrastructure of a capital is most of the times better than in regional and smaller cities. Due to the low mobility of homeless people and a frequent word-of-mouth recommendation which reaches only certain communities the health centre likely shows a limited target audience. Further, low-threshold care services for homeless people differ in terms of specific target groups or the scope of (medical) services offered. Reasons are a wild growth of providers, sources of funding or location. Extending the current design to additional health care facilities would contribute to insure findings and reduce the risk of selection bias. Nonetheless, as described earlier the included health care centre is kind of a local ‘maximum provider’ for homeless and uninsured patients. Among the homelessness community the health centre is well known and many shelters and accommodations across Berlin often refer homeless people to the centre. Thus, findings of the present study will contribute to a general better understanding of the health situation and health needs of homeless people and provide impetus beyond local health policy makers. Further, the study’s design and methods including the strength of analysing secondary data collected under naturalistic conditions may serve future health research in hard to reach populations.

Second, due to the unique health and social system in Germany the generalizability of findings will be constrained. This limitation has to be taken into account when interpreting results and comparing them to other health systems and countries. The German health system is not a universal healthcare system. Condition for access to medical services is the existence of a regular health insurance. However, despite standard statutory medical care, there are non-statutory welfare organisations that provide low-threshold universal care, especially for the homeless population. The Jenny De la Torre health centre represent such a low-threshold and free of charge care provider. It plays a key role in the medical care of their homeless patients, as it provides complementary universal care by primary physicians as well as medical specialists from different medical fields and in emergency cases, works together with hospitals in the surrounding area. Therefore, homeless individuals visit the centre for medical advice and care, for both, a short (acute symptoms) or a long (chronical conditions) period of time.

## Conclusion

In conclusion, the GIG study will provide a unique and essential insight into social and health care needs of homeless individuals. The results will provide impetus on how to improve the social and health situation of homeless people for both policy makers and health care providers. We regard this research project as an important study for providing knowledge on standardised assessment of homeless peoples social and health conditions. This might be useful for adapting current public health reporting for this vulnerable group. Further, based on the results, we aim to draw conclusions and discuss implications.

## Data Availability

Data is stored in a non-publically available repository. Data are however available from the corresponding author on request.
